# Depression among Adolescents of Rural Nepal: A Community-Based Study

**DOI:** 10.1155/2021/7495141

**Published:** 2021-02-04

**Authors:** Prayas Gautam, Maginsh Dahal, Harishchandra Ghimire, Sakuntala Chapagain, Kushalata Baral, Rohit Acharya, Sudip Khanal, Anu Neupane

**Affiliations:** ^1^School of Public Health, Chitwan Medical College, Chitwan, Nepal; ^2^Department of Public Health, Asian College for Advance Studies, Purbanchal University, Satdobato, Lalitpur, Nepal; ^3^Department of Community Medicine and Public Health, Chitwan Medical College, Chitwan, Nepal; ^4^Department of Nursing, Chitwan Medical College, Chitwan, Nepal; ^5^Department of Public Health, Nobel College, Pokhara University, Sinamangal, Kathmandu, Nepal; ^6^Population Services International/Nepal, Western Regional Hub, Nepalgunj, Nepal; ^7^Department of Public Health, Little Buddha College of Health Science, Minbhavan, Kathmandu, Nepal

## Abstract

This community-based study is aimed at finding out the prevalence of depression, and its correlates among adolescents of rural Nepal. Data were collected among adolescents after multistage stratified proportionate random sampling by using the Patient Health Questionnaire (PHQ-9) to assess the depression among adolescent. Chi-square test and logistic regression model were applied. The prevalence of depression was found to be 27%. Adolescents who were not satisfied with their academic performance were 2.4 times more likely to have the risk of depression (AOR = 2.417, CI: 1.097-5.326). Likewise, tobacco users were almost fourteen times (AOR = 13.858, CI: 2.090-91.90), who intended to harm themselves were two times (AOR = 2.323, CI: 1.078-5.005), sleep deprivation were fifteen folds (AOR = 14.911, CI: 7.376-30.145), often scolded by their parents was almost three times (AOR = 2.931, CI: 1.195-7.436), and having poor relationship with friends were 2.4 times more likely (AOR = 2.371, CI: 1.078-5.215) of having depression. Sleep deprivation has a long-term negative impact on health leading depression.

## 1. Introduction

Adolescence aged 10 to 19 years is a critical period for the achievement of social emotional capabilities which would be the foundation for future life, health, and wellbeing. Helpful and supportive environments in the family, schools, and communities empower adolescents to maintain good mental health and wellbeing, but adolescents are also in the period of vulnerability for the onset of mental health conditions [1]. Depression is a common mental disorder and one of the main causes of disability globally. Depression is the ninth leading cause of illness and disability in all adolescents (WHO). Depression is a foremost contributor to the global burden of disease and often starts at a young age (WHO).

Globally, an estimated 300 million people are affected by depression [2]. Evidences show that depression is considered a major risk factor for increasing premature death among adolescent [3]. Adolescent depressive symptoms are emerging as a public health problem, mostly in developed countries as well as in developing countries [2]. Depression often starts in young age and has negative consequences in adolescent's health [4]. The highest prevalence of adolescent suicide is evidenced in Southeast Asia and Eastern Europe [3]. It can be persistent, comprehensively impairing adolescent's ability to function at work or school and to cope with daily life [5, 6]. Literatures have revealed that sleeping disorder may cause psychiatric disorders and psychological stress leading to acute and chronic depression [7]. Sleep deprivation in adolescence period is a risk for depression and suicide and increased substance abuse [8].

Mental health problems are estimated to affect 10-20% of children and adolescent worldwide, accounting 15-30% of disability-adjusted life years (DALYs) lost during first three decades of life [9]. Poor mental health can have negative effects on health and development of adolescents. It is associated with several health and social outcomes such as higher alcohol, tobacco and drug use, adolescent pregnancy, school dropout, and involvement in criminal behaviour (WHO).

Mental health problems in young people are not only a major public health challenge but are also a development issue in low- and middle-income countries. Recognizing and addressing depression of young people help them function better socially, academically, and vocationally and develop into well-adjusted and productive adults is prime [9]. Various researches explained that sleep deprivation among adolescents is one of the major contributor for depression [10, 11].

Evidence on the burden of depression among adolescents in Nepal is not well measured due to the lack of adequate research [5, 6]. In Nepal, mental health services receive insignificant attention at all levels of society, from the government to the general public. This is despite studies showing that mental health problems account for a significant proportion of the global burden of disease [12].

This study will help us to shed light on the present situation of adolescent depression. Thus, we conducted the study to find out the prevalence of depression and its associated factors among adolescents of Nepal.

## 2. Methods

A descriptive cross-sectional study was conducted among the adolescent students of age 15-19 years currently studying in grade 11 and 12 of rural Nepal.

### 2.1. Sample Size and Sampling Technique

The sample size was determined by using the prevalence of 21.2% (prevalence was obtained from a study conducted in Nepal among undergraduate students) [13].

The sample size was determined by using a formula.

Sample size for finite population (*n*) = *n*_0_/(1 + (*n*_0−1_/*N*)) [14].

Then, the sample size for infinite population (*n*_0_) = *z*^2^*pq*/*d*^2^ [14].

Where *n* is the minimum required sample size. *z* is the critical value on standard normal distribution at 95% confidence interval (1.96). *p* is the prevalence of self-medication (34.4%). *d* is the margin of error desired around *p* to be estimated (5%/0.05). *n* = 1.962∗0.344∗(1 − 0.656)/0.05^2^. *n* = 347.

Additional 7% sample was added for nonresponse. Thus the final sample size was 371.

Multistage stratified proportionate random sampling was done among 7 public and 9 private schools. The total number of students in public was 3197 and private was 3342. Schools were selected in equal number, i.e., three schools from private and three schools from the public as the total number of students in both public and private was almost equal. The numbers of schools were selected by using probability proportionate to size (PPS). Then, in the second stage, the sample size from each school was determined proportionately, and then in the final stage, further stratification was done in class 11 and 12 and from each faculty (science, management, and education). The sample frame was prepared from the school attendance. The required sample of 371 was selected by random number table through excel. Adolescent students aged between 15-19 years who were present during the day of data collection were included in this study.

### 2.2. Data Collection Tools

Semistructured, self-administrated questionnaire was used for data collection. The questionnaire included sociodemographic information, behavioural, and psychological characteristics of students and Patient Health Questionnaire (PHQ-9) tool for assessing the depressive symptoms. The Patient Health Questionnaire (PHQ-9) is a self-administered version for the assessment of depression. PHQ-9 is a nine-item scale, in which score for each item of PHQ-9 ranges from 0 (not at all) to 3 (almost every day) based on the frequency of symptoms experienced. PHQ-9 score ranges from 0 to 27, in which a greater score reflects more depressive symptoms. A PHQ score ≥ 10 has a sensitivity of 88% and specificity of 88% and was used to measure the depressive symptoms [15].

### 2.3. Data Collection Procedure and Technique

The questionnaire was in Nepali language and piloted in 50 individuals before use in the survey. Translated version of PHQ-9 used in this study has undergone internal validity test using Chronbach's alpha, and the value was 0.75. Participants were briefed about the study objectives, and written parental consent was taken for the respondent age below 18 years. Data were collected in a separate classroom. Participants were briefed about the techniques of filling the questionnaire. Seating arrangements of the students were made properly in such a way that chances of peeking each other's answers were as low as possible.

### 2.4. Statistical Analysis

Data collected went through coding, editing, entry, and rechecking and were done by researcher's each day. Data entry and analysis were done using Epi-Data 3.1 and IBM SPSS version 20. Descriptive analysis was used to describe background characteristics and the prevalence of depression among adolescents. Data were reported in the form of proportion, means, and standard deviation. Chi-square test was used to test the difference between the categorical variables. Variables that were found statistically significant (*p* < 0.05) during bivariate analysis were checked for multicollinearity and then further analyzed using logistic regression model in multivariate analysis. Adjusted odds ratio with 95 percent CI and *p* value was calculated. Hosmer and Lemeshow test was used to test the goodness-of-fit for the regression model. Predictability of regression and coefficient of determination (Nagelkerke R square) for the equation were calculated. Multicollinearity test has been done to find out the possibility of multicollinearity among independent variables. To identify whether there exists serious collinearity problem, variance inflation factor (VIF) has been estimated which is reciprocal to tolerance.

## 3. Results

Table 1 portrays the demographical and economical characteristics of 371 respondents. Mean age (in years) of the respondents was 17.39 (SD ± 0.92). Almost three-fifths of the participants (59%) were from advantaged caste group (Brahmin/Chhetri). 89.2 percent of the students were following Hindu religion, and almost four-fifth (79.3%) were receding in urban area. Majority (88.1%) of the students live in nuclear family, and more than three-fifth (63.5%) have a family size of up to five. Majority (92.2%) of the parents were living together. More than one third (38%) of the family monthly income was up to twenty thousand.

Table 2 presents characteristics of respondent's parents. One third (33.91%) of respondent's father have studied more than basic level but only less than one fifth (18.41%) of respondent's mother have studied more than eighth grade. Most of the student's parents have agriculture (41.0%) as major occupation. Almost two-fifth (39.37%) of the respondent's fathers had alcohol but majority (95.6%) of respondent's mother never had alcohol.

Table 3 represents the educational characteristics of the students. 53.6 percent of the respondents were from grade 11, almost equal students were enrolled in data collection in public and private institution. More than one third (39.6%) were studying in management faculty. Four fifth (80.6%) of the students currently studying their respective faculty were because of their own preference and more than three quarters (78.1%) were passed in their previous exams. About two-third (63.6%) of the students was not satisfied with their academic performance.

Table 4 represents the behavioural characteristics of respondents. Among 371 students, majority (94.1%) of the students were nontobacco users, and 94.3% of students have never had alcohol. More than three-fifths of the students occasionally do physical exercise. 56.3% of the respondents participated in extracurricular activities conducted by their schools. Almost half of the respondents used the internet up to two hours daily, and more than one-fifth of the respondents used the internet for more than four hours of internet. More than half (54.5%) of the respondents used a minimum of 1 hour of internet before going to bed.

Table 5 portrays the psychological factors of respondents. More than three quarters (78.7%) report they have had conflict in their families. Great majority (92.5%) of the parents never scold their children. Almost half (49.3%) of the students had a good relationship with friends, and about one third (30.5%) students had a good relationship with teachers as well. More than a quarter (28.9%) students have either girlfriend or boyfriend. More than one third (38.8%) of the respondents felt lonely. Less than one-tenth (7.6%) of the students have left their home without informing their parents regardless of the frequency of running away from home. More than one fifth (21.6%) of the students had tried to hurt themselves irrespective to the frequency of hurting themselves. Students frequently share their feeling more with their close friends than with family.

**Figure 1:**
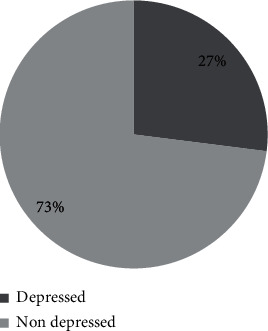
Prevalence of depression among respondents (*n* = 371).

Figure 1 depicts the prevalence of depression among adolescents. Respondents had a mean PHQ-9 score of 7.35 (SD ± 4.04). More than one quarter (27%) of the respondents were depressed.

Table 6 shows the multicollinearity by the VIF factor. None of them have tolerance < 0.1 and VIF > 10. There was no problem of collinearity among independent variables as the highest VIF was 1.765.

The results from multivariable linear regression models of the relationship of students' characteristics with sleep deprivation, internet addiction, and depressive symptoms are tabled in Table 7. Those respondents who were not satisfied with their academic performance were 2.4 times more likely to have risk of depression than those students who were satisfied with their academic performance (AOR = 2.417, CI: 1.097-5.326). Tobacco users were almost fourteen times more likely to be depressed than their counterparts (AOR = 13.858, CI: 2.090-91.90). Those students who intended to harm themselves were two times more likely to develop depression (AOR = 2.323, CI: 1.078-5.005). Those students who have sleep deprivation were fifteen folds in risk developing depression than students without sleep deprivation (AOR = 14.911, CI: 7.376-30.145). Those students who were often scolded by their parents were almost three times more likely to be depressed than those students whose parent never scolds (AOR = 2.931, CI: 1.195-7.436). Similarly, those students who have poor relationship with friends were 2.4 times more likely to be in risk of depression than those students who have a good relationship with their friends (AOR = 2.371, CI: 1.078-5.215).

## 4. Discussion

We found that mean family size was 5.51 (± 2.12 SD) which was higher than National 4.6 [16]. In this study, the prevalence of depression was found to be 27%. Prevalence of depression among adolescent students as reported from existing literature varies from (6.4% to 52.9%) [13, 17–22]. A study with similar setting in Bangladesh among secondary school children found the prevalence was 25%, which was similar to our research findings [20]. Prevalence was found a little bit lower in a study conducted by Bhandari et al. [13]. However, Guo et al. found the prevalence very low compared to our findings, i.e., 6.4% [18]. The difference may be due to difference in sample size in this study was very low compared to that study, and the tools used in to measure depression different research were different. Study conducted by Sandal et al. in India reported prevalence was found comparatively higher than ours which was 36.1, and study conducted in Iran also reported a higher prevalence than ours [17, 22]. Malik et al. showed the highest prevalence of depression among adolescent students of Hariyana, India, which was 52.9%, and another study conducted in Bihar, India have also similar prevalence, 49.2% [19, 21].

In this study, very few sociodemographic variables have a significant association with depression. Age, sex, ethnicity, family type, family size, parent marital status, monthly income occupation, occupation of father, and occupation of mother were found insignificant with depression among students. However, sex was found to be significantly associated with depression with female more at risk of developing depression in studies conducted in Brazil and Malaysia [23, 24]. Similar to our findings, age was found insignificant in the study conducted by Singh et al. in Chandigarh North India [25]. Educational status of parents and drinking status of parents were found to be associated with depression in this study. Those students whose parents were educated more than basic levels were more likely to develop depression than those students whose parents were less educated. The finding could be attributed to the fact that educated parents have higher expectation from children. Apart from that, they often try to indulge their children more toward academic activities and often much of their time of enjoyment or recreational activities get reduced. So when compared with educated parents who are often involved in a job or other activities, the less educated parents are more available to their children to share their feelings and thoughts, thereby reducing the risk of depression. However, a study conducted in Iran does not show any significant association between parents' level of education and depression among children [17]. In contrary with this findings, a study conducted in Malaysia, those students whose parents are less educated were more likely to be depressed [26].

In this study grade, reason for selecting the faculty, achievement in previous exam, and satisfaction with academic performance was found to be statistically significant with depression, whereas the type of school and faculty of students were not found significant with depression of students. This study revealed that those students studying in higher grades, i.e., 12 standard were 1.7 times more likely to have risk of depression than their counterparts (OR = 1.676, CI: 1.056-2.659), and a study conducted by Singh et al. in India shows similar findings, where highest grade students were 1.6 times more likely to be depressed than those students from lowest grade (OR = 1.659, CI: 0.228-12.099) [25]. In this study, those students who do not have their own decision for selecting currently studying faculty were more likely to have depressive symptoms than those students who selected their subject with their own decision. The findings could be attributed to the fact that those students who selected their career as a peer, their interest could feel less pressure in studies, could do well in their studies, and could be more satisfied with their academic life, thereby reducing stress and risk of depression. In a similar study in India, those students whose parents choose their career without their children decision were more likely to be depressed than their counterparts [25]. Similarly, those students who failed in their previous exams were more likely to be at risk of depression than those students who have passed their previous exam. Study conducted by Bhandari et al. also shows the similar findings [13]. Satisfaction with academic performance was also associated with depression among adolescent students. Those students who were not satisfied with academic performance were more than two times more likely to be depressed than those students who were satisfied with their academic performance (OR = 2.417, CI: 1.097-5.326). Similar findings were reported in a study conducted in India (OR = 5.089, CI: 11.180-21.957) [25].

Tobacco use and alcohol use were found to be statistically significant with depression among adolescent students, whereas physical activity and participation in extracurricular activities were not found significant with depression. But a study conducted in Korea revealed that those adolescents with no physical activity were found to be a risk factor for depression [27]. In these findings, tobacco use increases the risk of developing depression by almost 7 folds (OR = 6.655, CI: 2.626-16.866), and a study conducted among adolescent of Brazil also showed similar significant with depression (OR = 2.23, CI: 1.12-4.43) [24]. Similarly, alcohol-user students were 2.6 times more likely to be depressed than their counterparts (OR = 2.626, 1.079-0.639). Likewise, significant association was found in a study conducted in Brazil, where alcohol users were on more risk of developing depression (OR = 2.22, CI: 1.21-4.07).

Conflict in family, having a girlfriend or boyfriend, and feeling lonely were found to have no relationship with developing risk of depression among adolescents. The findings could attribute to the fact that having a girlfriend or boyfriend could help in sharing thoughts and feelings with each other, thereby reducing the risk of depression. Scolding by family members often was significantly associated with depression among adolescent students in this study. The alike findings were reported from a study conducted in India [25]. Relationship with a teacher and relationship with a friend were also a risk factor for developing depression among adolescents according to our findings. However, a study conducted among Chinese adolescents found no significant association between relationship with teacher and risk factor for depression [18]. Having poor relationship with classmates was a risk factor for depression (AOR = 2.371, CI: 1.078-5.215), and alike findings were reported in a study conducted in China (AOR = 1.60, CI: 1.14-2.25) [18]. In this study, run away from home without informing their parents and tried to hurt them were also significantly associated with depression. Study conducted among Chinese students reported significant association between run away from home with depression but no significant association between tried to hurt themselves on purpose and risk of developing depression [18].

Having smartphone and use of internet were not found to be significant with risk of developing depression but more use of internet on a daily basis and use of the internet before falling asleep was found to be statistically associated with developing risk of depression among students. Those students who used the internet for more than an hour before falling asleep were two times more likely to develop depression than those who never used the internet before sleeping. Students who used the internet for more than four hours daily were most likely to be depressed than those who never use the internet (OR = 3.412, CI: 1.419-8.205). Likewise, longer daily time use of internet was analyzed as a risk factor for depression in a study conducted by Lim et al. in Korea. Those students using the internet more than three hours a day were two times more likely to have increased risk of depression (OR = 2.235, CI: 1.078-4.637) [27]. Similarly, another study conducted in India shows the use of social sites was associated with developing risk of developing depression [25].

Sleep deprivation was statistically significant with depression among students in this study. Evidences from other studies clearly show sleep deprivation and depression are strongly interrelated. Those students who have sleep deprivation have a strong effect on the risk of developing depression [10, 24, 28–30]. The odds of increasing risk of developing depression almost increase by five folds in a student with sleep deprivation according to study conducted by Roberts et al. in Texas [10]. In this study, those students lacking proper sleep or having sleep deprivation were fifteen times more likely to have depression than those students with normal sleep (OR = 15.041, CI: 8.397-26.939). Similar findings were reported in Brazil where the odds of developing risk of depression increase by ten times among poor sleepers (OR = 10.440, CI: 1.400-46.070) [24]. The findings of our study provide the baseline information for the policymakers so that they could not deny the existence of problem in Nepalese community. Our finding shows that there might be a possible association between the various factors and depression among the adolescents but it cannot establish the causal relationships. We suggest the broader spectrum of similar kind of study in large population on interventional setting for the clear picture of the real situation.

## 5. Conclusion

We can conclude that the problem of depression among adolescents is like an iceberg tip in Nepalese context. This study reflects the clear picture in a small arena and cannot be generalized but can show the policymakers and stakeholders that the problems exist in our context. Improved sleep quality will likely to benefit Nepalese school students in their mental health status, daily activities, and academic performance.

## 6. Limitations

Since the study is conducted among adolescent and in a small area, it cannot be generalized to the whole population all over Nepal. School dropout adolescent and those adolescents who were currently not studying were excluded from the sample.

## Figures and Tables

**Table 1:** Demographic and economic characteristics of respondents (*n* = 371).

Characteristics	*n* (%)
Age mean age ± SD (years)	17.39 (SD ± 0.92)
Gender	
Male	189 (50.9)
Ethnicity	
Brahmin/Chhetri	219 (59)
Religion	
Hindu	331 (89.2)
Place of residence of the respondent	
Urban	292 (79.3)
Type of family (*n* = 370)	
Nuclear	252 (68.1)
Family size	
≤5	235 (63.5)
Parent living status	
Living together	342 (92.2)
Monthly income of family (in NRs)	
≤20,000	141 (38)
>20,000—≤25,000	76 (20.5)
>25,000—≤30,000	81 (21.8)
>30,000	73 (19.7)

**Table 2:** Distribution of characteristics of family of respondents' (*n* = 371).

Characteristics	*n* (%)
Educational status of father (*n* = 348)	
≤8	230 (66.1)
>8	118 (33.9)
Educational status of mother (*n* = 364)	
≤8	297 (81.6)
>8	67 (18.4)
Occupational status of father (*n* = 346)	
Government job	62 (17.9)
Private job	47 (13.5)
Business	61 (17.6)
Agriculture	142 (41.0)
Others^∗^	34 (9.8)
Occupational status of mother (*n* = 362)	
Government job	16 (4.4)
Private job	34 (9.3)
Business	45 (12.4)
Agriculture	209 (57.7)
Others^∗∗^	58 (16.0)
Drinking status of father (*n* = 348)	
No	211 (60.6)
Drinking status of mother (*n* = 364)	
No	348 (95.6)

^∗^Includes foreign employment and labour work. ^∗∗^Includes housewife, labour work, and foreign job.

**Table 3:** Educational characteristics of the respondents (*n* = 371).

Characteristics	*n* (%)
Grade	
11	209 (56.3)
12	162 (43.7)
Type of school	
Public	183 (49.3)
Private	188 (50.7)
Faculty of the respondent	
Science	112 (30.2)
Management	147 (39.6)
Education	112 (30.2)
Reason for selecting the currently studying faculty	
Own decision	299 (80.6)
Others^∗^	72 (19.4)
Achievement in last exam (*n* = 370)	
Passed	290 (78.1)
Fail	81 (21.9)
Satisfied with academic performance	
Yes	236 (36.4)
No	135 (63.6)

^∗^Includes failing to qualify in other faculty, family pressure, and friends pressure.

**Table 4:** Behavioural characteristics of respondents (*n* = 371).

Characteristics	*n* (%)
Tobacco users	
No	349 (94.1)
Alcohol users	
No	350 (94.3)
Do physical exercise	
Regularly	46 (12.4)
Frequently	37 (10)
Occasionally	237 (63.9)
Rarely	31 (8.4)
Never	20 (5.4)
Participate in extracurricular activities	
Yes	209 (56.3)
Daily time spent on internet (hours)	
Never	46 (12.4)
>0–≤2	151 (46.5)
>2–≤4	132 (40.6)
>4	42 (12.9)
Daily time spent on internet (hours) before going bed (*n* = 325)	
Never use	22 (6.8)
>0—≤1	177 (54.5)
>1	126 (38.7)

**Table 5:** Description of psychological factors of respondents (*n* = 371).

Characteristics	Percentage
Conflict in family	
Yes	293 (78.7)
Family members scold you	
No	343 (92.5)
Relationship with friends	
Good	183 (49.3)
Average	128 (34.5)
Poor	60 (16.2)
Relationship with teacher	
Good	113 (30.5)
Average	169 (45.6)
Poor	89 (24.0)
Do you have girlfriend or boyfriend	
No	265 (71.4)
Felt lonely	
No	227 (61.2)
Run away from home	
No	343 (92.5)
Hurt yourself	
No	291 (78.4)
Sharing with parents (*n* = 370)	
Frequently	78 (21.1)
Occasionally	258 (69.7)
Rarely	23 (6.2)
Never	11 (3.0)
Sharing with friends	
Frequently	150 (40.4)
Occasionally	181 (48.8)
Rarely	30 (8.1)
Never	10 (2.7)

**Table 6:** Test of multicollinearity of different independent variables related to depression.

Model (constant)	Tolerance	VIF
Grade of the student	0.951	1.052
Reason for selecting currently studying faculty	0.823	1.215
Achievement in last exam	0.607	1.649
Satisfied with academic performance	0.567	1.765
Tobacco use	0.620	1.613
Family members scold you often	0.836	1.196
Relationship with teacher	0.697	1.435
Relationship with friends	0.725	1.379
Run away from home	0.727	1.376
Hurt yourself	0.796	1.256
Time spend on internet	0.606	1.650
Spend time on internet before going to bed	0.605	1.654
Sleep deprivation	0.869	1.151
Drinking status of father	0.845	1.183
Drinking status of mother	0.923	1.084
Education status of father	0.655	1.527
Education status of mother	0.644	1.553

**Table 7:** Multivariable linear regression of factors associated with depression.

Characteristics	UOR (95% CI)	AOR (95% CI)	*p* value
Grade (11)	1.676 (1.056-2.659)	1.675 (0.881-3.187)	0.116
Reason for selecting current faculty (own decision)	3.159 (1.8466-5.406)	1.712 (0.778-3.769)	0.182
Achievements in last exam (pass)	2.675 (1.590-4.499)	1.121 (0.460-2.735)	0.801
Satisfied with academic performance (yes)	3.920 (2.424-6.338)	2.417 (1.097-5.326)	0.029^∗^
Tobacco use (yes)	6.655 (2.626-16.866)	13.858 (2.090-91.90)	0.006^∗^
Run away from home (yes)	4.841 (2.180-10.749)	1.075 (0.337-3.424)	0.903
Tried to hurt yourself (yes)	2.902 (1.724-4.884)	2.323 (1.078-5.005)	0.031^∗^
Time spend on internet (never)			
>0–≤2	1.381 (0.563-2.673)	1.480 (0.256-8.558)	0.662
>2–≤4	2.252 (0.926-5.476)	0.658 (0.092-4.698)	0.677
>4	8.193 (2.974-22.571)	1.167 (0.142-9.594)	0.886
Time spend on internet before going to bed (never)			
≤1	1.232 (0.576-2.673)	1.322 (0.283-6.181)	0.723
>1	5.167 (2.396-10.887)	4.679 (0.851-25.728)	0.761
Sleep deprivation (yes)	15.041 (8.397-26.939)	14.911 (7.376-30.14)	0.0001^∗^
Education status of father (≤8)	1.671 (1.026-2.723)	1.775 (0.935-3.366)	0.079
Education status of mother (≤8)	1.860 (1.061-3.262)	1.313 (0.621-2.776)	0.475
Drinking status of father (no)	1.935 (1.198-3.125)	1.643 (0.963-2.773)	0.069
Drinking status of mother (no)	3.799 (1.374)	3.056 (0.986-9.527)	0.054
Family members scold you (no)	5.729 (2.544-12.903)	2.931 (1.195-7.436)	0.024^∗^
Relationship with friend (good)			
Poor	3.122 (1.677-5.812)	2.371 (1.078-5.215)	0.032^∗^
Average	1.436 (0.847-2.345)	1.416 (0.762-2.636)	0.271
Relationship with teachers (good)			
Poor	3.264 (1.733-6.149)	1.445 (0.649-3.217)	0.367
Average	1.403 (0.778-2.532)	2.047 (0.519-2.114)	0.897

Characteristics mentioned in brackets resembles reference category. UOR: unadjusted odds ratio; AOR: adjusted odds ratio; ^∗^Include statistically significant; Cox & Snell *R*^2^ = 0.365, Nagelkerke *R*^2^ = 0.531, and Hosmer and Lemeshow = 0.154.

## Data Availability

The datasets used and/or analyzed during the current study are available from the corresponding author on reasonable request.

## Abbreviations

AOR: Adjusted odds ratio
BMC: Bio med central
CI: Confidence interval
CMC: Chitwan Medical College
DALY: Disability-adjusted life years
DAS: Depression and anxiety scale
DPHO: District Public Health Office
IOM: Institute of Medicine
OR: Odds ratio
PHQ-9: Patient Health Questionnaire-9
PPS: Probability proportionate to size
PSQI: Pittsburgh Sleep Quality Index
PSS: Perceived Stress Scale
SD: Standard deviation
SPSS: Statistical Package for Social Sciences
VIF: Variance inflation factor
WHO: World Health Organisation

## Additional Points

*Code Availability*. The software application or custom codes used in the research are available from the corresponding author (MD) upon reasonable request.
